# Oral Tolerance: Therapeutic Implications for Autoimmune Diseases

**DOI:** 10.1080/17402520600876804

**Published:** 2006

**Authors:** Ana M. C. Faria, Howard L. Weiner

**Affiliations:** 1 Departamento de Bioquímica e Imunologia Instituto de Ciências Biológicas Universidade Federal de Minas Gerais Belo Horizonte MG Brazil; 2 Harvard Medical School Center for Neurologic Diseases Brigham and Women's Hospital Boston MA USA

## Abstract

Oral tolerance is classically defined as the suppression of immune responses to antigens (Ag) that have been administered previously by the oral route. Multiple mechanisms of tolerance are induced by oral Ag. Low doses favor active suppression, whereas higher doses favor clonal anergy/deletion. Oral Ag induces Th2 (IL-4/IL-10) and Th3 (TGF-β) regulatory T cells (Tregs) plus CD4+CD25+ regulatory cells and LAP+T cells. Induction of oral tolerance is enhanced by IL-4, IL-10, anti-IL-12, TGF-β, cholera toxin B subunit (CTB), Flt-3 ligand, anti-CD40 ligand and continuous feeding of Ag. In addition to oral tolerance, nasal tolerance has also been shown to be effective in suppressing inflammatory conditions with the advantage of a lower dose requirement. Oral and nasal tolerance suppress several animal models of autoimmune diseases including experimental allergic encephalomyelitis (EAE), uveitis, thyroiditis, myasthenia, arthritis and diabetes in the nonobese diabetic (NOD) mouse, plus non-autoimmune diseases such as asthma, atherosclerosis, colitis and stroke. Oral tolerance has been tested in human autoimmune diseases including MS, arthritis, uveitis and diabetes and in allergy, contact sensitivity to DNCB, nickel allergy. Positive results have been observed in phase II trials and new trials for arthritis, MS and diabetes are underway. Mucosal tolerance is an attractive approach for treatment of autoimmune and inflammatory diseases because of lack of toxicity, ease of administration over time and Ag-specific mechanism of action. The successful application of oral tolerance for the treatment of human diseases will depend on dose, developing immune markers to assess immunologic effects, route (nasal versus oral), formulation, mucosal adjuvants, combination therapy and early therapy.


*Clinical & Developmental Immunology*, June–December 2006; 13(2–4): 143–157

Taylor & Francis

Taylor & Francis Group

### Oral tolerance: Therapeutic implications for autoimmune diseases

ANA M. C. FARIA[1] & HOWARD L. WEINER[2]

[1]*Departamento de Bioquímica e Imunologia, Instituto de Ciências Biológicas, Universidade Federal de Minas Gerais, Av. Antonio Carlos, 6627, Belo Horizonte, MG 31270-901, Brazil, and* [2]*Harvard Medical School, Center for Neurologic Diseases, Brigham and Women's Hospital, 77 Avenue Louis Pasteur, Boston, MA 02115, USA*

**Abstract**

Oral tolerance is classically defined as the suppression of immune responses to antigens (Ag) that have been administered previously by the oral route. Multiple mechanisms of tolerance are induced by oral Ag. Low doses favor active suppression, whereas higher doses favor clonal anergy/deletion. Oral Ag induces Th2 (IL-4/IL-10) and Th3 (TGF-β) regulatory T cells (Tregs) plus CD4+CD25+ regulatory cells and LAP+T cells. Induction of oral tolerance is enhanced by IL-4, IL-10, anti-IL-12, TGF-β, cholera toxin B subunit (CTB), Flt-3 ligand, anti-CD40 ligand and continuous feeding of Ag. In addition to oral tolerance, nasal tolerance has also been shown to be effective in suppressing inflammatory conditions with the advantage of a lower dose requirement. Oral and nasal tolerance suppress several animal models of autoimmune diseases including experimental allergic encephalomyelitis (EAE), uveitis, thyroiditis, myasthenia, arthritis and diabetes in the nonobese diabetic (NOD) mouse, plus non-autoimmune diseases such as asthma, atherosclerosis, colitis and stroke. Oral tolerance has been tested in human autoimmune diseases including MS, arthritis, uveitis and diabetes and in allergy, contact sensitivity to DNCB, nickel allergy. Positive results have been observed in phase II trials and new trials for arthritis, MS and diabetes are underway. Mucosal tolerance is an attractive approach for treatment of autoimmune and inflammatory diseases because of lack of toxicity, ease of administration over time and Ag-specific mechanism of action. The successful application of oral tolerance for the treatment of human diseases will depend on dose, developing immune markers to assess immunologic effects, route (nasal versus oral), formulation, mucosal adjuvants, combination therapy and early therapy.

**Keywords:** *Oral tolerance, nasal tolerance, autoimmune diseases, bystander suppression*

### Autoimmunity and oral tolerance

"Immunological tolerance" has often been defined as a mechanism by which the immune system prevents pathologic autoreactivity against self and thus prevents autoimmune diseases. This definition of tolerance as a negative counterpart of immunity comes from the classical work of Burnet who first proposed self/non-self discrimination as a major principle driving the operation of the immune system and tolerance to self-components as a deletional event taking place at early periods of development (Burnet 1959). According to Burnet's "Clonal Selection Theory", self tolerance was based on the blindness of the mature immune system to body components. The description of the thymic selection of T lymphocytes, of the subsets of T cells and their distinct actions, as well as the demonstration of autoreactive B and T cells in normal individuals contributed to change this scenario (Avrameas 1991; Kerlero de Rosbo et al. 1993; Zhang et al. 1994; Lacroix-Desmazes et al. 1998). It became clear to several researchers that natural tolerance to autocomponents is a more complex phenomenon. Physiological autoimmunity, as a self-assertion process that generates immune regulation and pathological autoimmunity has to be distinguished as different outcomes of self-recognition (Cohen and Young 1991;

Correspondence: A. M. C. Faria, Departamento de Bioquímica e Imunologia, Instituto de Ciências Biológicas, Universidade Federal de Minas Gerais, Av. Antonio Carlos, 6627, Belo Horizonte, MG 31270-901, Brazil.

ISSN 1740-2522 print/ISSN 1740-2530 online © 2006 Taylor & Francis
DOI: 10.1080/17402520600876804

Modigliani et al. 1996). Studies derived from this new approach clearly demonstrated that natural tolerance to self is an active process that depends on the activity of non-inflammatory/regulatory auto-reactive T lymphocytes present at a stable frequency in the normal repertoire (Cohen and Young 1991; Modigliani et al. 1996; Sakaguchi 2004).

In analogy to the natural non-inflammatory reactivity that the immune system mounts to self components, the name "oral tolerance" has been given in the seventies (Vaz et al. 1977) to the immunological tolerance to antigens (Ag) that access the body via the oral route. Oral tolerance has been classically defined as the specific suppression of cellular and/or humoral immune responses to an Ag by prior administration of the Ag by the oral route. Since most of the natural contact with foreign Ag occurs via the mucosal surfaces, tolerance induction to commensal bacteria and dietary proteins represents the major immunological event taking place in the gut in physiological conditions.

Oral tolerance is of unique immunologic importance since it is a continuous natural immunologic event driven by exogenous Ag. Due to their privileged access to the internal milieu, commensal bacteria and dietary Ag that continuously contact the mucosa represent a frontier between foreign and self-components. Thus, oral tolerance is a form of peripheral tolerance that evolved to treat external agents that gain access to the body via a natural route as internal components that then become part of self.

Within the view of self tolerance as an active process, auto-immune diseases would arise from defects in immunoregulatory processes. The usual therapies for these pathological conditions rely on non-specific immunossupression with several undesirable side effects. Since oral tolerance is such a potent way of inducing regulatory cells towards specific Ag, the idea of using the oral route to trigger tolerance to Ag involved in autoimmune diseases comes as an important clinical application of the phenomenon. Although the idea already present as a theoretical possibility in the seventies, it was only successfully tested in the classical studies of four groups of researchers working on experimental models of arthritis (Nagler-Anderson et al. 1986; Thompson and Staines 1986) and multiple sclerosis (Bitar and Whitacre 1988; Higgins and Weiner 1988) in the eighties.

### Mechanisms of oral tolerance induction

A concept has arisen in oral tolerance studies that there are two primary effector mechanisms of oral tolerance: low doses of Ag favor the generation of regulatory cell-driven tolerance whereas high doses of Ag favor either clonal deletion (Chen et al. 1995; Marth et al. 1996; Meyer et al. 2001) or anergy of specific T cells (Mowat et al. 1982; Whitacre et al. 1991; Melamed and Friedman 1993; Friedman and Weiner 1994; Inada et al. 1997). Low doses of Ag would preferentially induce regulatory T cells (Tregs) that secrete down-modulatory cytokines such as TGF-β, IL-10 and IL-4 (Miller et al. 1992). TGF-β secreting $CD4^+$ and $CD8^+$ T cells have been isolated and cloned from the PP and MLN of orally tolerized mice (Chen et al. 1994, 1996; Santos et al. 1994; Wang et al. 1994) and from the peripheral blood of humans fed MBP (Fukaura et al. 1996). T cells cloned from tolerized mice have been ascribed to a unique subset of $CD4^+$ T cell, the Th3 cell (Chen et al. 1994; Fukaura et al. 1996; Faria and Weiner 2005). This population appears to be dependent on IL-4, rather than IL-2 for its growth and some Th3 clones produce IL-4 and/or IL-10 together with TGF-β (Chen et al. 1994; Faria and Weiner 2005). In addition, in TCR transgenic mice, oral adminstration of Ag also resulted in a relative increase of CD4+CD25+ Tregs expressing either CTLA-4 (Zhang et al. 2001) or FoxP3 (Mucida et al. 2005). These cells have suppressive properties *in vitro* and can transfer tolerance to naive recipients (Zhang et al. 2001; Nagatani et al. 2004). They either secrete high levels of IL-10 and TGF-β (Zhang et al. 2001) or are dependent in TGF-β for their development (Mucida et al. 2005). In addition to the secreted form of TGF-β, murine CD4+CD25+ or CD4+CD25 − regulatory cells have been reported to express latency-associated peptide (LAP) and TGF-β on the surface after activation and exert regulatory function by the membrane-bound TGF-β *in vitro* (Nakamura et al. 2001; Oida et al. 2003). Recombinant latency-associated peptide (rLAP) was also shown to reverse suppression by mouse CD4+CD25+ T cells as well as their human counterparts, $CD4+CD25^{high}$ T cells (Nakamura et al. 2004). Thus, surface expression of the latent form of TGF-β can be another mode of action for TGF-β.

The generation of Ag-specific regulatory cells seem to be a result of Ag presentation by gut-associated Antigen-presenting cells (APC) (Viney et al. 1998; Simioni et al. 2004) that are particularly involved in the induction of TGF-β-producing T cells (Simioni et al. 2004). These Ag-specific regulatory cells migrate to lymphoid organs suppressing immune responses by inhibiting the generation of effector cells and to target organs, suppressing disease by releasing Ag-non specific cytokines (bystander suppression).

Since these pioneer studies, evidence has been reported that the two forms of tolerance are not mutually exclusive and they may overlap. Recent studies on the properties of Tregs also describe them as "anergic" (Takahashi et al. 1998; Taams et al. 2002; Sakaguchi 2004) and deletional events taken place in the gut mucosa (Ramachandran et al. 2000) result local TGF-β secretion by macrophages

(Fadok et al. 1998) and dying T cells (Chen et al. 2001). This cytokine acts as a growth factor for the generation of Th3 cells (Paul and Seder 1994; Weiner 2001; Faria and Weiner 2005) and of CD4+FoxP3+ Tregs (Chen et al. 2003; Horwitz et al. 2003; Sakaguchi 2004). Thus, anergy/deletion and active regulation may be different aspects of the same tolerogenic processes that are triggered by antigenic contacts in the intestine.

### Major issues involved in the use of oral tolerance as a therapy

Oral tolerance has been already successfully tested in a number of experimental models for autoimmune diseases as well as other inflammatory conditions such as allergy and transplantation (Faria and Weiner 2005). After this intensive research work, three major issues emerged on the therapeutic use of oral tolerance for autoimmune diseases: (1) the choice of a target Ag; (2) the limiting therapeutic situation of treating already sensitized subjects; and (3) the large doses that are sometimes required for its efficient induction.

First, the rationale for the use of oral tolerance as a therapeutic tool in autoimmune conditions is mainly that it would provide a type of specific suppression avoiding the side effects of non-specific immunosupression. One potential problem in its use is the choice of the target Ag. It is not always clear which are the autoantigens involved in autoimmune pathogenesis. Fortunately, as we will discuss below, oral tolerance is associated with a bystander suppression event that spreads the regulatory activity triggered by oral tolerance to other Ag presented in the same context. Cross-suppression (Vaz et al. 1981) or bystander suppression (Miller et al. 1991) of a non-related inflammatory event by mucosally administered Ag is now a well described phenomenon that provides a way to circumvent the need of unique target for oral tolerance induction.

Second, it has been already reported that oral tolerance is easily induced in naïve animals but primed animals are more resistant to its suppressive effects (Yoshino et al. 1995; Conde et al. 1998; Faria and Weiner 2005). Indeed, immunized mice are susceptible to oral tolerance induction when the Ag is administered up to 7 days after immunization. Then, they become progressively resistant to tolerance induction and 14 days later they are completely refractory (Conde et al. 1998). As a way to circumvent this problem, there are a number of adjuvants and regimens of feeding already described that modulate positively the mechanisms involved in oral tolerance induction (see section on modulation of oral tolerance).

Third, to achieve effective suppression with the oral tolerance regimen, sometimes large doses of Ag are necessary. This requirement may hinder the use of oral tolerance to human subjects. As we will discuss below, nasal tolerance may be an alternative in many cases since the nasal mucosa is also an effective route to induce suppression and the doses required are usually 10-fold lower.

### Bystander suppression (Table I)

Bystander suppression is a concept that regulatory cells induced by a fed Ag can suppress immune responses stimulated by different Ag, as long as the fed Ag is present in the anatomic vicinity (Faria and Weiner 2005). It was described during an investigation of the regulatory cells induced by oral administration of low doses of MBP (Miller et al. 1991). It solves a major conceptual problem in the design of Ag- or T-cell-specific therapy for inflammatory autoimmune diseases such as MS, type 1-diabetes and rheumatoid arthritis (RA) in which the autoantigen is unknown or where there are reactivities to multiple autoantigens in the target tissue. During the course of chronic inflammatory autoimmune processes in animals, there is intra- and interantigenic spread of autoreactivity at the target organ (Lehmann et al. 1992; Cross et al. 1993; Tisch et al. 1993). Similarly in human autoimmune diseases, there are reactivities to

Table I. Models of autoimmune and other diseases that demonstrate bystander suppression.

| Disease | Immunizing Ag | Oral Ag | Target organ |
|---|---|---|---|
| Arthritis | BSA, mycobacteria | CII | Joint |
| EAE | PLP | MBP | Brain |
| EAE | MBP peptide 71–90 | MBP peptide 21–40 | Brain |
| EAE | MBP | OVA | Lymph node, DTH response |
| Diabetes | LCMV | Insulin | Pancreatic islets |
| IBD | CD4 + CD45RB$^{high}$ T cell transfer | OVA | Intestine |
| Stroke | None | MBP, MOG | Brain |
| Nerve injury | None | MBP | Brain |
| Atherosclerosis | None | Hsp65 | Aortic arch |

Abbreviation: BSA, bovine serum albumin; DTH, delayed-type hypersensitivity; EAE, experimental allergic encephalomyelitis; LCMV, lymphocytic choriomeningitis virus; MBP, myeline basic protein, OVA, ovalbumin; PLP, proteolipid; and IBD, inflammatory bowl disease.

multiple autoantigens in the target tissue. For example, in MS, there is immune reactivity to at least three myelin Ag: MBP, proteolipid protein (PLP) and myelin oligodendrocyte glycoprotein (MOG) (Kerlero de Rosbo et al. 1993; Zhang et al. 1994). In type 1 diabetes, there are multiple islet-cell Ag that could be the target of autoreactivity including glutamic acid decarboxylase (GAD), insulin and heat shock proteins (HSP) (Harrison 1992). Because regulatory cells induced by oral Ag secrete non-specific cytokines after being triggered by the fed Ag, they suppress inflammation in the microenvironment where the fed Ag is localized. Thus, for a human organ-specific inflammatory disease, it is not necessary to know the specific Ag that is the target of an autoimmune response, but only to feed an Ag capable of inducing regulatory cells, which then migrate to the target tissue and suppress inflammation.

The mechanisms by which this process could occur are usually assumed to reflect the production of inhibitory cytokines by the tolerized Treg, with resulting suppression of the T cells with other specificities in the vicinity. IL-10 and TGF β are the most favored cytokines in this respect. This idea is supported by several studies that showed TGF-β-dependent suppression in experimental allergic encephalomyelitis (EAE) studies using different myelin Ag (Miller et al. 1991, 1993). On the other hand, the role of IL-10 in the phenomenon is supported by the findings that OVA-specific Tr1 cells can prevent IBD (induced by gut bacteria), providing OVA is present in the intestinal environment (Groux et al. 1997).

Inhibitory mediators or molecules on the surface of Tregs could act directly on the responding T cells or they could act by "deactivating" the APC which is attempting to stimulate the third party T cell. Ag-presenting dendritic cells (DCs) can act as "temporal bridges" to relay information from orally tolerized memory T cells to naive CD4 + T cells (Lanzavecchia 1998). Since APC, specially Dcs, recirculate, this idea is consistent with all the data available on the phenomenon including reports showing that a type of bystander suppression occurs with Ag at different anatomic sites or injected days apart from each other (Carvalho et al. 1994, 1997).

Bystander suppression has been demonstrated in a number of autoimmune disease models. For instance, PLP-induced EAE can be suppressed by feeding MBP (al-Sabbagh et al. 1994) or by administering TGF-β-secreting MBP-specific T-cell clones from orally tolerized animals (Chen et al. 1994). In the Lewis rat model of EAE, disease induced by MBP peptide 71–90 can be suppressed by feeding peptide 21–40 (Miller et al. 1993). In arthritis models, adjuvant- and Ag-induced arthritis can be suppressed by feeding type II collagen (CII) (Yoshino et al. 1995). In a virus-induced model of diabetes, whereby a lymphocytic choriomeningitis virus (LCMV) protein is expressed on the insulin promoter and the animal is then infected with the LCMV, diabetes can be suppressed by feeding insulin (von Herrath et al. 1996).

In theory, bystander suppression could be applied for the treatment of organ-specific inflammatory conditions that are not classic autoimmune diseases, such as psoriasis, or could be used to target anti-inflammatory cytokines to an organ where inflammation may play a role in disease pathogenesis even if the disease is not primarily inflammatory in nature. For example, oral MBP decreased stroke size in a rat stroke model, presumably by decreasing inflammation associated with ischaemic injury (Becker et al. 1997). Induction of nasal tolerance in mice to a peptide of the myelin Ag MOG also reduces ischemic injury following stroke. Regulatory cells are generated in tolerant wild type but not in IL-10-deficient mice. Moreover, IL-10-producing MOG specific CD4+ T cells can transfer tolerance to naïve recipients (Frenkel et al. 2003, 2005). Bystander suppression achieved by oral Ag in non-immune pathological conditions may mimic a physiological protective reaction to self Ag in response to inflammatory insult as observed in the experimental model of central nervous system (CNS) axonal injury in EAE-susceptible and resistant rat strains. Oral treatment with low-dose MBP is beneficial for post-traumatic survival of retinal ganglion cells in Lewis rats following optic nerve injury (Monsonego et al. 1996). HSP, known to be up-regulated in inflammatory situations, are also a suitable target for bystander suppression strategies. Oral as well as nasal administration of hsp65 in LDL-R deficient mice fed a high colesterol diet downmodulates IL-2 and IFN-γ production and aortic plaque development. Production of IL-10 but not TGF-β is up-regulated in hsp-tolerized mice (Harats et al. 2002; Maron et al. 2002a).

### Modulation of oral tolerance (Table II)

Data on animal models showed also that a number of factors have been reported to modulate oral tolerance. As oral tolerance has usually been defined in terms of Th1 responses, anything that suppress Th1 and/or enhances Th2 or Th3 cell development would enhance oral tolerance. Th3 cells appear to use IL-4 and TGF-β itself as one of its growth/differentiation factors. Thus, IL-4 administration i.p., oral IL-10 and IL-4 can also enhance oral tolerance when co-administered with Ag and cytokines have also been administered by the nasal route (Inobe et al. 1998; Slavin et al. 2001). Oral but not subcutaneous lipopolysaccaride (LPS) enhances oral tolerance to MBP and is associated with increased expression of IL-4 in the brain (Khoury et al. 1992). In the uveitis model, intraperitoneal IL-2 potentiates oral tolerance and is associated with increased production of TGF-β, IL-10 and IL-4 (Rizzo et al. 1994). Oral Ag delivery

Table II. Modulation of oral tolerance

| Enhances | Prevents |
|---|---|
| IL-2 | IFN-γ |
| IL-4, IL-10 | IL-12, IL-18 |
| Helminth Ag | CD80 over-expression |
| Anti-CD4 | Anti-TGF-β Ab |
| Anti-IL-12 | CT |
| CTB | Anti-MCP-1 (CCL2) |
| LPS | IBD |
| Flt3L | Anti-γδ Ab |
| Type 1 IFN (β and τ) | GVHR |
| Multiple emulsions | CY, 2′-dGuo |
| Lipossomes | Oestradiol |
| PLGA | |
| Continuous feeding | |
| TGF-β | |
| Pooled IgG | |

Abbreviations: CT, cholera toxin; CTB, cholera toxin B subunit; IFN, interferon; IL, interleukin; LPS, lipopolysaccharide; MCP, monocyte chemotactic protein 1; GVHR, Graft-versus-Host Reaction; CY, cyclophosphamide; 2′-dGuo, 2′-deoxyguanosine; TGFβ, transforming growth factor? β; and PLGA, poly lactic-co-pucolic acid.

using either a multiple emulsion system (Kim et al. 2002; Pecquet et al. 2000) or liposomes (Masuda et al. 2002) or poly-lactic-co-pucolic acid (PLGA) (Kim et al. 2002) also enhances oral tolerance. In the arthritis model, administration of TGF-β or dimaprid (a histamine type 2 receptor agonist) i.p., both of which are believed to promote the development of immunoregulatory cells, enhances the induction of oral tolerance to collagen II even after the onset of arthritis (Thorbecke et al. 1999). Coupling Ag to recombinant cholera toxin B subunit (CTB) enhances their ability to induce peripheral immune tolerance (Holmgren et al. 2003). A recently reported vaccine consisting of a fusion protein composed of CTB subunit and insulin produced in the hemolymph of silkworm showed ability to enhance oral tolerance induction in non-obese diabetic mice (Gong et al. 2005). On the other hand, cholera toxin (CT) is one of the most potent mucosal adjuvants and feeding CT abrogates oral tolerance when fed with an unrelated protein Ag. Large doses of IFN-γ given intraperitoneally abrogate oral tolerance (Zhang et al. 1990a), anti-IL-12 enhances oral tolerance and is associated both with increased TGF-β production and T cell apoptosis (Marth et al. 1996) and subcutaneous administration of IL-12 reverses mucosal tolerance (Eaton et al. 2003). Oral IFN-β and IFN-τ (tau) synergizes with the induction of oral tolerance in SJL/PLJ mice fed low doses of MBP (Nelson et al. 1996; Soos et al. 2002). Other exogenous agents which have been reported to enhance oral tolerance when given orally include parasite Ag from *H. polygyrus* (Shi et al. 2000), polysaccharide AZ9 from *Klebsiella oxitoca* (Sugihara et al. 2002) and *Schistosoma Mansoni* egg antigens (SEA) (Maron et al. 1998). Antibody (Ab) to chemokine monocyte chemotactic protein 1 (MCP-1) abrogates oral toleran (Karpus et al. 1996). Intraperitoneal co-adminstration of normal IgG to mice orally treated with Ag leads to a sustained and intense immunological tolerance including those of lupus-prone NZB X NZW lineage (Mengel et al. 2005). We also found recently that not only agents but certain regimens of feeding interfere with oral tolerance induction. Continuous and serial feeding regimens of the Ag administration enhances significantly oral tolerance by up-regulating IL-10 and TGF-β (Faria et al. 2003).

### Nasal tolerance (Table III)

In the nasal and upper respiratory tract of human and animals, there is an intact mucosal lymphoid tissue containing two structures: nasal associated lymphoid tissue (NALT) and the bronchus associated lymphoid tissue (BALT). In rodents, there is a lymphoid tissue that surrounds the nasal cavity whereas in humans and certain other species, there is an oropharyngeal lymphoid tissue (Waldeyer's ring) that includes the adenoid and the bilateral tubule, palatine and lingual tonsils (Goeringer and Vidic 1987). The bronchial mucosas resemble the gut mucosa with a network of Dcs and lymphocytes scattered in the airway epithelium. One relevant difference between the absorption of Ag in the upper respiratory tract and in the gut is the absence of digestion in the former. This feature may explain why minute doses of Ag are effective in inducing tolerance via the nasal route as compared to the high doses usually required for oral administration.

Nasal adminstration of Ag has been shown to suppress a number of experimental autoimmune diseases including diabetes in nonobese diabetic (NOD) mice (Daniel and Wegman 1996; Harrison et al. 1996; Tian et al. 1996), pristane- and collagen-induced arthritis (Staines et al. 1996; Myers et al. 1997; Garcia et al. 1999; Lu and Holmdahl 1999), EAE in mice (Metzler and Wraith 1993; Liu et al. 1998) and rats (Bai et al. 1997; Li et al. 1998), experimental autoimmune myasthenia gravis (EAMG) (Ma et al. 1995; Shi et al. 1999), experimental

Table III. Autoimmune and inflammatory disease models suppressed by nasal tolerance.

| Model | Administered protein |
|---|---|
| EAE | MBP, MBP peptides |
| EAMG | AChR |
| EAU | Retinal Ag |
| Diabetes | Insulin, GAD65 |
| EAN | Myelin peptide |
| Arthritis | Hsp65, collagen II and IX, CII peptide |
| Atherosclerosis | Hsp65 |
| Stroke | MOG peptide |

autoimmune uveitis (EAU) (Dick et al. 1993) and experimental autoimmune neuritis (EAN) (Zhu et al. 1998).

The mechanisms involved in the inhibition of disease development after nasal administration of Ag involve induction of CD4+T cell unresponsiveness or Treg development (Harrison et al. 1996; Shi et al. 1999; Akbari et al. 2001; Samsom et al. 2004). These Tregs are described as γβ CD8+ T cells (Harrison et al. 1996) or ab CD4+T cells that secrete high amounts of IL-10 (Akbari et al. 2001; Samsom et al. 2004). Akbari et al. (2001) suggest that IL-10-producing DCs from the lungs are particularly effective in inducing the development of Tr1 cells (CD4+ cells that produce IL-10) upon nasal administration of Ag. Some authors also found nasal tolerance to be associated with the induction of TGF-β-producing T cells (Shi et al. 1999; Ostroukhova et al. 2004). Interestingly, bystander suppression in experimental models of atherosclerosis (Maron et al. 2002a,b) and stroke (Frenkel et al. 2003, 2005) is effectively induced by nasal administration of Ag and suppression it is mediated by IL-10-producing T cells.

### Therapeutic applications of oral tolerance in autoimmune and inflammatory diseases in animals (Table IV)

Several studies have demonstrated the effectiveness of oral administered autoantigens in animal models of autoimmune and inflammatory diseases (Faria and Weiner 2005).

### *Experimental allergic encephalomyelitis*

The first studies to show that orally administered myelin Ag could suppress EAE were performed in the Lewis rat. EAE was suppressed by low doses of oral MBP and MBP fragments (Higgins and Weiner 1988) and by high doses of MBP given in bicarbonate (Bitar and Whitacre 1988). High doses of MBP can suppress EAE via the mechanism of T cell clonal anergy (Javed et al. 1995) whereas multiple lower doses prevent EAE by transferable active cellular suppression (Miller et al. 1993). In the nervous system of low-dose-fed animals, inflammatory cytokines such as TNF and IFN-γ are down-regulated and TGF-β is up-regulated (Khoury et al. 1992). Administration of myelin to sensitized animals in the chronic guinea pig model or larger doses of MBP in the murine EAE model is protective and does not exacerbate disease (Brod et al. 1991; Meyer et al. 1996) and long term (6 month) administration of myelin in the chronic EAE model was beneficial (al-Sabbagh et al. 1996a,b).

A number of studies have demonstrated suppression of EAE in murine models. Both conventional and T cell receptor transgenic animals have been used and both oral MBP and oral PLP have been administered although the majority of studies have used MBP. In these models, MBP regulatory clones have been described and such cells have also been induced in MBP T cell receptor transgenic mice. Both CD4 and CD8 cells have been shown to mediate active suppression and anergy/deletion have also been demonstrated in oral tolerance to EAE.

The latest approach in animal models has been to utilize glatiramer acetate (Cop1, Copaxone), a drug approved for therapy of multiple sclerosis which is given to patients by injection. Teitelbaum et al. (1999) have found that oral glatiramer acetate suppresses EAE in both the mouse and rat models and we have found that oral glatiramer acetate suppresses EAE in MBP T cell receptor transgenic animals and induces the upregulation of TGF-β when given orally (Maron et al. 2002b). Our working hypothesis is that glatiramer acetate is acting as an altered peptide ligand and is immunologically active in the gut (Weiner 1999).

Table IV. Experimental models of autoimmune diseases suppressed by oral tolerance.

| Model | Protein fed |
|---|---|
| EAE | MBP, PLP, MOG, copaxone |
| Arthritis (CIA, AA, AIA, PIA, SCW) | Collagen II, Hsp65, BSA |
| Diabetes (NOD mouse) | Insulin, insulin β-chain, GAD, OVA |
| Colitis | Haptenized or normal colonic proteins, OVA |
| EAU | S-Ag, IRBP |
| EAMG | AchR |
| Anti-phospholipid syndrome | β2-Glycoprotein |
| Experimental allergic neuritis | PNS-myelin |
| Thyroiditis | Thyroglobulin |
| Atherosclerosis | Hsp65 |
| Stroke | MOG |
| Nerve injury | MBP |

### *Arthritis models*

There are several animal models of arthritis including collagen induced arthritis (CIA), adjuvant arthritis (AA), pristane induced arthritis (PIA), antigen-induced arthritis (AIA), silicone induced arthritis and streptococcal cell with arthritis.

Immunization with heterologous or homologous species of CII produces autoimmune responses to CII that lead to development of arthritis in susceptible mouse strains (Courtenay et al. 1980). CIA has been used as an animal model for RA and is characterized by chronic inflammation within the joints, associated with synovitis and erosion of cartilage and bone (Trentham et al. 1977). The first experiments of oral tolerance using rat CIA was done by Thompson and Staines (1986) and Nagler-Anderson et al. (1986) in WA/KIR rats and DBA/1 mice, respectively by immunizing with CII either in complete or in incomplete Freund's adjuvant (CFA). Feeding of CII prior to immunization delayed the onset and suppressed the incidence of CIA.

Collagen peptides are also capable of inducing CIA. Immunodominant collagen peptides have been used to suppress CIA by oral administration. Khare et al. (1995) induced CIA to DBA/1 mice by immunizing with human CII peptide (250–270) in CFA. Oral tolerance with human peptide CII (250–270) abolished anti-human and anti-mouse CII Ab and markedly reduced the disease severity both at early and effector phases.

Another major model for RA is AA, which is a well-characterized and fulminant form of experimental arthritis. Oral administration of chicken CII consistently suppressed the development of AA in Lewis rats and the suppression of AA could be adoptively transferred by T cells from CII fed animals (Zhang et al. 1990b).

HSP plays an important role in the AA model (van Eden et al. 1988). It was recently reported that oral administration of mycobacterial 65-kDa HSP suppressed the development of AA in rats (Haque et al. 1996). Suppression of AA was adoptive transferred by spleen cells from orally tolerized rats.

Oral tolerance is also effective in PIA (Thompson et al. 1993). Immunizing mice twice with 2, 6, 10, 14-tetramethylpentadecane (pristane) twice leads to arthritis after 100 ~ 200 days. Increasing doses of oral CII lowered both the incidence and severity of PIA.

Other animal models of arthritis that have been successfully treated by mucosal tolerance include streptococcal cell wall arthritis (Chen et al. 1998) and silicone induced arthritis (Yoshino 1995).

Methotrexate is a widely used drug in RA. We found that there is a synergistic effect between methotrexate and orally administered Ag such as MBP (al-Sabbagh et al. 1997) and a synergistic effect with oral methotrexate was also observed in the AA model (Weiner and Komagata 1998).

*Diabetes*

Oral insulin has been shown to delay and in some instances, prevent diabetes in the NOD mouse model. Such suppression is transferable (Zhang et al. 1991), primarily with $CD4^+$ cells (Bergerot et al. 1999). Immunohistochemistry of pancreatic islets of Langerhans isolated from insulin fed animals demonstrates decreased insulitis (Hancock et al. 1995). Oral insulin suppressed diabetes in a viral induced model of diabetes in which LCMV was expressed under the insulin promoter and animals infected with LCMV to induce diabetes (von Herrath et al. 1996). Protection was associated with protective cytokine shifts (IL-4/IL-10, TGF-β) in the islets. It has also been shown that expression of TGF-β in the pancreatic islets protects the NOD mouse from diabetes (King et al. 1998). Oral administration of β-chain of insulin, a 30-amino-acid peptide slowed the development of diabetes and prevented diabetes in some animals (Polanski et al. 1997). This effect was associated with a decrease IFN-γ and an increase in IL-4, TGF-β and IL-10 expression. Oral dosing of bacterial stimulants such as LPS and *E. Coli* extract OM-89 in NOD mice induces a Th2 shift in the gut cytokine gene expression and concomitantly, improves diabetes prevention by oral insulin administration (Bellmann et al. 1997). Oral administration of recombinant GAD from plant sources suppressed the development of diabetes in NOD mouse as does oral administration of a plant-based CTB-insulin fusion protein (Arakawa et al. 1998).

*Colitis*

TGF-β appears to play a crucial role in the development of animal models of colitis, including TNBS-colitis, colitis in the IL-2-deficient animal model following systemic administration of TNP-KLH in adjuvant and in the model of Th1 colitis in SCID mice. It has been shown that TNBS colitis can be prevented by oral administration of TNBS, which acts via the induction of TNBS-specific TGF-β responses (Neurath et al. 1996). TNBS-induced colitis in rats can also be prevented by feeding either human colon epithelial cells or rat colonic epithelial extracts, but not human fibroblasts nor rat small intestine extracts, showing that tolerance is organ specific. Protected animals had low IFN-γ and high TGF-β levels and tolerance could be transferred by mesenteric lymph nodes cells (Dasgupta et al. 2001). In addition, colitis can be suppressed via bystander suppression by transfer of OVA transgenic CD4+-$CD45^{high}$ T cells (from DO.11.10 mice) into SCID mice (Zhou et al. 2004).

*Uveitis*

Oral administration of S-Ag, a retinal autoantigen that induces EAU, or S-Ag peptides prevents or markedly diminishes the clinical appearance of S-Ag-induced disease as measured by ocular inflammation (Nussenblatt et al. 1990). S-Ag-induced EAU can also be suppressed by feeding an HLA peptide (Wildner and Thurau 1994). Feeding interphotoreceptor binding protein (IRBP) suppresses IRBP-induced disease and is potentiated by IL-2 (Rizzo et al. 1994). Oral feeding of retinal Ag not only can prevent acute disease but also can effectively suppress second attack in chronic-relapsing EAU, demonstrating that oral tolerance may have practical clinical implications in uveitis, which is predominantly a chronic-relapsing condition in humans (Thurau et al. 1997a,b). Other investigators have found that oral administration of bovine S-Ag peptides is very efficient in preventing EAU but could only inhibit mild disease if feeding was delayed until after immunization and relatively high feeding doses were required (Ma et al. 1998; Torseth and Gregerson 1998).

*Myasthenia*

Although myasthenia gravis is an Ab-mediated disease, oral administration of the Torpedo acetylcholine receptor (AchR) to Lewis rats prevented or delayed the onset of myasthenia gravis. The levels of anti-AchR antibodies in the serum were lower in orally tolerized animals than in control animals. The effect was dose dependent and large doses of Ag (at least 5 mg of AchR) plus soybean tripsin inhibitor (STI) (Wang et al. 1993) were required, suggesting that anergy may be the primary mechanism. Purified AchR was found more effective than an unpurified mixture (Okumura et al. 1994).

*Other autoimmune diseases*

The antiphospholipid syndrome is characterized by the presence of high titers of IgG anticardiolipin antibodies and/or lupus anticoagulant antibodies. Oral administration in BALB/c mice of low doses of β2 glycoprotein prevented the serologic and clinical manifestation of experimental antiphospholipid syndrome upon immunization with the autoantigen. Decreased T cell responses, Ab responses and increased expression of TGF-β which mediated the suppression was demonstrated. Tolerance was transferred by CD8 positive class I restricted TGF-β secreting cells (Blank et al. 1998).

Immune complex disease can be suppressed both following administration of a single large dose of Ag (Browning and Parrott 1987) or by placing Ag in drinking water (Devey and Bleasdale 1984). These studies were performed before oral tolerance was applied to autoimmune diseases. It has also been raised whether defective oral tolerance may be associated with experimental IgA nephropathy (Gesualdo et al. 1990). A recent report shows that feeding of Ro 274 peptide or Ro 60 to BALB/c mice with Sjogren's syndrome-like disease (immunized with Ro 60 previously) drastically reduces salivary infiltrates and specific Ab production (Kurien et al. 2005).

Thyroiditis has been effectively suppressed following oral administration of either porcine or human thyroglobulin (Guimarães et al. 1995; Peterson and Braley-Mullen 1995). In the murine model, CD8 positive regulatory cells which produce IL-4 and TGF-β mediated the suppression (Guimaraes et al. 1995). These cells also appear to induce bystander suppression upon triggering with the fed Ag. Other investigators (Zhang and Kong 1998) reported that tolerance induced by IV administration of deaggregated thyroglobulin in experimental autoimmune thyroiditis (EAT) is dependent on CD4+ T cells but independent of IL-4/IL-10.

Experimental allergic neuritis is the counterpart of EAE and can be suppressed both by oral administration of peripheral nerve proteins (Gaupp et al. 1997).

Link's group has experimented with administering Ag associated with more than one autoimmune disease and immunizing with a mixture of Ag. They have demonstrated that oral administration of AChR plus MBP suppresses EAMG and EAE (Wang et al. 1995). Thus, it appears there is no interference by one autoantigen versus another when they are from different target organs.

### Treatment of autoimmune diseases in humans using oral tolerance (Table V)

Based on the long history of oral tolerance and the safety of the approach, human trials have been initiated in autoimmune diseases, MS, RA, uveitis and diabetes. These initial trials suggest that there has been no systemic toxicity or exacerbation of disease, although clinical efficacy resulting in an approved drug has yet been demonstrated. Results in humans however, have paralleled several aspects of what has been observed in animals.

Table V. Human studies of oral tolerance application in autoimmune diseases.

A. Diabetes
- Preliminary report: Preserved beta-cell function as measured by endogenous C-peptide in new onset diabetics over 20 years old fed 1 mg;
- Oral insulin not beneficial in new onset disease fed 2.5 or 7.5 mg oral insulin for 1 year;

B. Multiple sclerosis
- Oral myelin decreased MRI lesions in DR2 + males, no effect on clinical relapse;
- Increased TGF-β secreting myelin cells after oral myelin;

C. RA
- Oral collagen ameliorates RA;
- Oral collagen benefits juvenile RA in open label trial;
- 20 μg best in double blind oral dosing trial of CII;
- Oral bovine collagen at higher doses without positive effect;
- 60 μg oral collagen best in composite analysis of dosing trials; no different than placebo in phase III trial;
- Bovine collagen (0.5 mg) beneficial in placebo controlled trial;
- Oral collagen II beneficial in JRA clinically and immunologically in pilot trial;

D. Uveitis
- Oral S-Ag appeared to allow medication taper, retinal mixture appeared to worsen uveitis;
- Oral HLA peptide allowed steroid taper;

E. Thyroid disease
- Decreased cellular immunity to thyroglobulin in patients receiving oral thyroglobulin

*Diabetes*

Six different trials are currently underway or completed in testing mucosally administered recombinant human insulin as a tolerizing agent in type 1 diabetes: (1) a multicenter double-blind study in the US evaluating oral insulin therapy versus placebo in adults and children with new-onset disease. Preliminary analysis suggests preserved beta cell function as measured by endogenous C-peptide insulin responses in patients diagnosed over age 20 years and fed 1 mg. versus placebo (Ergun-Longmire et al. 2004); (2) a double-blind study in France to compare oral insulin therapy and parenteral insulin therapy versus placebo in patients during the remission phase. Evaluation criteria include duration of remission, measures of insulin secretion/sensitivity and immunological parameters; no effect of treatment with 2.5 or 7.5 mg oral insulin was observed (Chaillous et al. 2000); (3) a multicenter double-blind study in Italy to evaluate whether the addition of oral insulin is able to improve the integrated parameters of metabolic control and modify immunological findings compared to placebo in patients with recent onset disease treated with intensive insulin therapy (IMDIAB VI). No effect of subcutaneous insulin was observed but a positive effect of 7.5 mg of oral insulin in a high risk cohort was observed and a repeat trial of 7.5 mg of oral insulin is being planned at patients at risk. (Skyler, J., Oral communication. Immunology of Diabetes Society Meeting, Cambridge, England, 2004); (4) a multicenter double-blind study in the US to determine if diabetes can be prevented by subcutaneous insulin therapy or oral insulin therapy in subjects at risk for diabetes (DPT-1); (5) a double-blind study in Australia evaluating aerosolized insulin versus placebo in patients with new-onset disease; and (6) a double-blind study in Finland evaluating nasally administered insulin versus placebo in patients with new-onset disease. A recent report shows that oral insulin treatment of patients with type 1 diabetes for 12 months was able to deviate the pattern of response of their peripheral mononuclear cells towards an increased production of TGF-β (Monetini et al. 2004).

*Multiple sclerosis*

In MS patients, MBP- and PLP-specific TGF-β-secreting Th3-type cells have been observed in the peripheral blood of patients treated with an oral bovine myelin preparation and not in patients who were untreated (Fukaura et al. 1996). These results demonstrate that it is possible to immunize via the gut for autoantigen-specific TGF-β-secreting cells in a human autoimmune disease by oral administration of the autoantigen. However, a completed 515 patient, placebo-controlled, double-blind phase III trial of single-dose bovine myelin in relapsing-remitting MS did not show differences between placebo and treated groups in the number of relapses; a large placebo effect was observed (AutoImmune Inc., Lexington, MA, USA). The dose of myelin was 300 mg given in capsule form and contained 8 mg MBP and 15 mg PLP. Preliminary analysis of magnetic resonance imaging data showed significant changes favoring oral myelin in certain patient subgroups. Trials in MS have been undertaken with the MBP analogue, glatiramer acetate, which is currently given by injection to MS patients but has been shown to be effective orally in animals and to induce regulatory cells that mediate bystander suppression (Teitelbaum et al. 1999; Weiner 1999). A phase III trial of oral glatiramer acetate given daily at 5 and 50 mg versus placebo found no clinical, MRI, or immunologic effects. Phase II trials testing oral GA at doses of 300 and 600 mg are currently in progress.

*Rheumatoid arthritis*

In RA, a 280 patient double-blind phase II dosing trial of CII in liquid doses ranging from 20 to 2500 μg per day for six months demonstrated statistically significant positive effects in the group treated with the lowest dose (Barnett and Kremer 1998). Oral administration of larger doses of bovine CII (1–10 mg) did not show a significant difference between tested and placebo groups, although a higher prevalence of responders was reported for the groups treated with CII. These results are consistent with animal studies of orally administered CII in which protection against adjuvant- and Ag-induced arthritis and bystander suppression was observed only at the lower doses (Zhang et al. 1990b; Yoshino 1995). An open-label pilot study of oral collagen in juvenile RA gave positive results with no toxicity (Barnett et al. 1996). This lack of systemic toxicity is an important feature for the clinical use of oral tolerance, especially in children for whom the long-term effects of immunosuppressive drugs is unknown.

Five phase II randomized studies of oral CII have been performed and based on the results obtained, a multicenter double-blind phase III trial study of oral CII (Colloral®) was undertaken (AutoImmune Inc.). In the five double-blind phase II studies a total of 805 patients were treated with oral CII and 296 treated with placebo. Two of the studies have been published (Trentham et al. 1993; Barnett et al. 1998). The other three studies were included in a integrated analysis that led to the decision to carry out a phase III trial. A dose refinement study tested doses of 5, 20 and 60 μg. Colloral at 60 μg was found to be the most significant dose compared to other doses. Safety analysis demonstrated that Colloral was extraordinarily safe with no side effects. The magnitude of the clinical responses of Colloral appears to be on the same level as NSAIDS for the majority of patients. However, there is a sub-group of patients that appear to have a more significant response to the medication. Based on these data, a 760-patient phase III trial was performed comparing 60 μg of Colloral to placebo. However, no differences were observed. There was a large placebo effect in the control group. Subsequently, a placebo controlled trial of bovine collagen showed significant effects in those receiving 0.5 mg, but not in groups receiving 0.05 or 5 mg (Choy et al. 2001). Oral CII in juvenile RA was associated with clinical improvement and decreased CII specific IFN-γ and increased TGF-β (Myers et al. 2001). Clinical trials are underway to determine whether withholding NSAIDS and prednisone will allow OT to be induced and whether oral CII has meaningful clinical efficacy in RA (Postlethwaite 2001).

### *Uveitis*

In uveitis, a pilot trial of S-Ag and an S-Ag mixture was conducted at the National Eye Institute (Bethesda, MD, USA) and showed positive trends with oral bovine S-Ag but not the retinal mixture (Nussenblatt et al. 1997). Feeding of peptide derived from patient's own HLA Ag appeared to have effect on uveitis in that patients could discontinue their steroids because of reduced intraocular inflammation mediated by oral tolerance (Thurau et al. 1997b).

### *Thyroid*

Thirteen patients receiving thyroid hormone replacement with synthetic thyroxin were randomly assigned to receive oral porcine thyroid or remain on synthetic T4 (Lee et al. 1998). Humoral and cellular immune responses were measured over the course of a year. A decrease in cellular immunity to thyroid peptides was observed in the fed versus the control group. No changes between groups were observed in autoantibody levels.

### *Other*

Positive effects were reported in an open label pilot study of oral type I collagen in patients with systemic sclerosis (McKown et al. 1997, 2000).

### Future directions

In spite of the negative results to date of phase III human trials of mucosal tolerance, many lessons have been learned from them as well as from the sucessful experiments with animal models. Based on the results of oral tolerance in uveitis in humans (Nussenblatt et al. 1997) and in animal models of myasthenia (Okumura et al. 1994) and EAE (Benson et al. 1999), it appears that protein mixtures may not be as effective oral tolerogens as purified proteins.

Although it is clear that oral Ag can suppress autoimmunity and inflammatory diseases in animals, it is now clear from all these studies that several factors need to be carefully considered to improve the effectiveness of oral tolerance in human disease: (1) the dose of Ag is crucial; (2) a clear immunological marker or immunological effect has to be established as a parameter for the follow-up of each disease; (3) some mucosal adjuvants that enhance induction of mucosal have been described and may need to be used; (4) purified proteins are more effective oral tolerogens than protein mixtures; (5) frequency of oral administration interferes with efficiency of suppression achieved; (6) combination therapy using conventional anti-inflammatory and immunosuppressive drugs may yield a better result; and (7) early therapy is an important factor since oral tolerance is mostly effective before or shortly after disease onset.

### References

Akbari O, DeKruyff RH, Umetsu DT. 2001. Pulmonary dendritic cells producing IL-10 mediate tolerance induced by respiratory exposure to antigen. Nat Immunol 2(8):725–731.

al-Sabbagh A, Miller A, Santos LMB, Weiner HL. 1994. Antigen-driven tissue-specific suppression following oral tolerance: Orally administered myelin basic protein suppresses proteolipid induced experimental autoimmune encephalomyelitis in the SJL mouse. Eur J Immunol 24:2104–2109.

al-Sabbagh A, Nelson PA, Akselband Y, Sobel RA, Weiner HL. 1996a. Antigen-driven peripheral immune tolerance: Suppression of experimental autoimmmune encephalomyelitis and collagen-induced arthritis by aerosol administration of myelin basic protein or type II collagen. Cell Immunol 171:111–119.

al-Sabbagh AM, Goad EP, Weiner HL, Nelson PA. 1996b. Decreased CNS inflammation and absence of clinical exacerbation of disease after six months oral administration of bovine myelin in diseased SJL/J mice with chronic relapsing experimental autoimmune encephalomyelitis. J Neurosci Res 45(4): 424–429.

al-Sabbagh AM, Garcia G, Slavin AJ, Weiner HL, Nelson PA. 1997. Combination therapy with oral myelin basic protein and oral methotrexate enhances suppression of experimental autoimmune encephalomyelitis. Neurology 48:A421.

Arakawa T, Yu J, Chong DK, Hough J, Engen PC, Langridge WH. 1998. A plant-based cholera toxin B subunit-insulin fusion protein protects against the development of autoimmune diabetes. Nat Biotechnol 16:934–938.

Avrameas S. 1991. Natural autoantibodies. From "horror autotoxicus" to "gnothi seauton". Immunol Today 12:154–159.

Bai XF, Shi FD, Xiao BG, Li HL, van der Meide PH, Link H. 1997. Nasal administration of myelin basic protein prevents relapsing experimental autoimmune encephalomyelitis in DA rats by activating regulatory cells expressing IL-4 and TGF-beta mRNA. J Neuroimmunol 80(1–2):65–75.

Barnett ML, Combitchi D, Trentham DE. 1996. A pilot trial of oral type II collagen in the treatment of juveline rheumatoid arthritis. Arthritis Rheum 4:623–628.

Barnett ML, Kremer JM, St Clair EW, Clegg DO, Furst D, Weisman M, Fletcher MJ, Chasan-Taber S, Finger E, Morales A, Le CH, Trentham DE. 1998. Treatment of rheumatoid arthritis with oral type II collagen. Results of a multicenter, double-blind, placebo-controlled trial. Arthritis Rheum 41:290–297.

Becker KJ, McCarron RM, Ruetzler C, Laban O, Sternberg E, Flanders KC, Hallenbeck JM. 1997. Immunologic tolerance to myelin basic protein decreases stroke size after transient focal cerebral ischemia. Proc Natl Acad Sci USA 94:10873–10878.

Bellmann K, Kolb H, Hartmann B, Rothe H, Rowsell P, Rastegar S, Burghardt K, Scott FW. 1997. Intervention in autoimmune diabetes by targeting the gut immune system. Int J Immunopharmacol 19:573–577.

Benson JM, Stuckman SS, Cox KL, Wardrop RM, Gienapp IE, Cross AH, Trotter JL, Whitacre CC. 1999. Oral administration of myelin basic protein is superior to myelin in suppressing established relapsing experimental autoimmune encephalomyelitis. J Immunol 162:6247–6254.

Bergerot I, Arreaza GA, Cameron MJ, Burdick MD, Strieter RM, Chensue SW, Chakrabarti S, Delovitch TL. 1999. Insulin B-chain reactive CD4+ regulatory T-cells induced by oral insulin treatment protect from type 1 diabetes by blocking the cytokine secretion and pancreatic infiltration of diabetogenic effector T-cells. Diabetes 48:1720–1729.

Bitar DM, Whitacre CC. 1988. Suppression of experimental autoimmune encephalomyelitis by the oral administration of myelin basic protein. Cell Immunol 112:364–370.

Blank M, George J, Barak V, Tincani A, Koike T, Shoenfeld Y. 1998. Oral tolerance to low dose beta 2-glycoprotein I: Immunomodulation of experimental antiphospholipid syndrome. J Immunol 161:5303–5312.

Brod SA, al-Sabbagh A, Sobel RA, Hafler DA, Weiner HL. 1991. Suppression of experimental autoimmune encephalomyelitis by oral administration of myelin antigens: IV. Suppression of chronic relapsing disease in the Lewis rat and strain 13 guinea pig. Ann Neurol 29:615–622.

Browning MJ, Parrott DM. 1987. Protection from chronic immune complex nephritis by a single dose of antigen administered by the intragastric route. Adv Exp Med Biol 216B:1619–1625.

Burnet M. 1959. The clonal selection theory of acquired immunity. Nashville, Tennessee: Vanderbilt University Press.

Carvalho CR, Verdolin BA, de Souza AV, Vaz NM. 1994. Indirect effects of oral tolerance in mice. Scand J Immunol 39:533–538.

Carvalho CR, Verdolin BA, Vaz NM. 1997. Indirect effects of oral tolerance cannot be ascribed to bystander suppression. Scand J Immunol 45:276–281.

Chaillous L, Lefevre H, Thivolet C, Boitard C, Lahlou N, Atlan-Gepner C, Bouhanick B, Mogenet A, Nicolino M, Carel JC, Lecomte P, Marechaud R, Bougneres P, Charbonnel B, Sai P. 2000. Oral insulin administration and residual beta-cell function in recent-onset type 1 diabetes: A multicentre randomised controlled trial. Diabete insuline orale group. Lancet 356:545–549.

Chen Y, Kuchroo V, Inobe J, Hafler DA, Weiner HL. 1994. Regulatory T cell clones induced by oral tolerance: Suppression of autoimmune encephalomyelites. Science 265:1237–1240.

Chen Y, Inobe J, Marks R, Gonnella P, Kuchroo VK. 1995. Peripheral deletion of antigen-reactive T cells in oral tolerance. Nature 376:177–180.

Chen Y, Inobe J, Kuchroo VK, Baron JL, Janeway CA Jr, Weiner HL. 1996. Oral tolerance in myelin basic protein T-cell receptor transgenic mice: Suppression of autoimmune encephalomyelitis and dose-dependent induction of regulatory cells. Proc Natl Acad Sci USA 93:388–391.

Chen W, Jin W, Cook M, Weiner HL, Wahl SM. 1998. Oral delivery of group A streptococcal cell walls augments circulating TGF-beta and suppresses streptococcal cell wall arthritis. J Immunol 161:6297–6304.

Chen W, Jin W, Tian H, Sicurello P, Frank M, Orenstein JM, Wahl SM. 2001. Requirement for transforming growth factor beta1 in controlling T cell apoptosis. J Exp Med 194:439–453.

Chen W, Jin W, Hardegen N, Lei KJ, Li L, Marinos N, McGrady G, Wahl SM. 2003. Conversion of peripheral CD4+ CD25− naive T cells to CD4+CD25+ regulatory T cells by TGF-beta induction of transcription factor Foxp3. J Exp Med 198: 1875–1886.

Choy EH, Scott DL, Kingsley GH, Thomas S, Murphy AG, Staines N, Panayi GS. 2001. Control of rheumatoid arthritis by oral tolerance. Arthritis Rheum 44:1993–1997.

Cohen IR, Young DB. 1991. Autoimmunity, microbial immunity and the immunological homunculus. Immunol Today 12(4):105–110.

Conde AA, Stransky B, Faria AM, Vaz NM. 1998. March Interruption of recently induced immune responses by oral administration of antigen. Braz J Med Biol Res 31(3):377–380.

Courtenay JS, Dallman MJ, Dayan AD, Martin A, Mosedale B. 1980. Immunisation against heterologous type II collagen induces arthritis in mice. Nature 283:666–668.

Cross AH, Tuohy VK, Raine CS. 1993. Development of reactivity to new myelin antigens during chronic relapsing autoimmune demyelination. Cell Immunol 146:261–269.

Daniel D, Wegmann DR. 1996. Protection of nonobese diabetic mice from diabetes by intranasal or subcutaneous administration of insulin peptide B-(9–23). Proc Natl Acad Sci USA. 93(2):956–960.

Dasgupta A, Kesari KV, Ramaswamy KK, Amenta PS, Das KM. 2001. Oral administration of unmodified colonic but not small intestinal antigens protects rats from hapten-induced colitis. Clin Exp Immunol 125:41–47.

Devey ME, Bleasdale K. 1984. Antigen feeding modifies the course of antigen-induced immune complex disease. Clin Exp Immunol 56:637–644.

Dick AD, Cheng YF, McKinnon A, Liversidge J, Forrester JV. 1993. Nasal administration of retinal antigens suppresses the inflammatory response in experimental allergic uveoretinitis. A preliminary report of intranasal induction of tolerance with retinal antigens. Br J Ophthalmol 77(3):171–175.

Eaton AD, Xu D, Garside P. 2003. Administration of exogenous interleukin-18 and interleukin-12 prevents the induction of oral tolerance. Immunology 108:196–203.

Ergun-Longmire B, Marker J, Zeidler A, Rapaport R, Raskin P, Bode B, Schatz D, Vargas A, Rogers D, Schwartz S, Malone J, Krischer J, Maclaren NK. 2004. Oral insulin therapy to prevent progression of immune-mediated (type 1) diabetes. N Y Acad Sci 1029:260–277.

Fadok VA, Bratton DL, Konowal A, Freed PW, Westcott JY, Henson PM. 1998. Macrophages that have ingested apoptotic cells *in vitro* inhibit proinflammatory cytokine production through autocrine/paracrine mechanisms involving TGF-beta, PGE2, and PAF. J Clin Invest 101:890–898.

Faria AM, Weiner HL. 2005. Oral tolerance. Immunol Rev 206:232–259.

Faria AM, Maron R, Ficker SM, Slavin AJ, Spahn T, Weiner HL. 2003. Oral tolerance induced by continuous feeding: Enhanced up-regulation of transforming growth factor-beta/interleukin-10 and suppression of experimental autoimmune encephalomyelitis. J Autoimmun 20:135–145.

Frenkel D, Huang Z, Maron R, Koldzic DN, Hancock WW, Moskowitz MA, Weiner HL. 2003. Nasal vaccination with myelin oligodendrocyte glycoprotein reduces stroke size by inducing IL-10-producing CD4+ T cells. J Immunol 171: 6549–6555.

Frenkel D, Huang Z, Maron R, Koldzic DN, Moskowitz MA, Weiner HL. 2005. Neuroprotection by IL-10-producing MOG CD4+ T cells following ischemic stroke. J Neurol Sci 233 (1–2):125–132.

Friedman A, Weiner HL. 1994. Induction of anergy or active suppression following oral tolerance is determined by antigen dosage. Proc Natl Acad Sci USA 91:6688–6692.

Fukaura H, Kent SC, Pietrusewicz MJ, Khoury SJ, Weiner HL, Hafler DA. 1996. Induction of circulating myelin basic protein and proteolipid protein-specific transforming growth factor-beta1-secreting Th3 T cells by oral administration of myelin in multiple sclerosis patients. J Clin Invest 98:70–77.

Garcia G, Komagata Y, Slavin AJ, Maron R, Weiner HL. 1999. Suppression of collagen-induced arthritis by oral or nasal administration of type II collagen. J Autoimmun 13(3): 315–324.

Gaupp S, Hartung HP, Toyka K, Jung S. 1997. Modulation of experimental autoimmune neuritis in Lewis rats by oral application of myelin antigens. J Neuroimmunol 79:129–137.

Gesualdo L, Lamm ME, Emancipator SN. 1990. Defective oral tolerance promotes nephritogenesis in experimental IgA nephropathy induced by oral immunization. J Immunol 145:3684–3691.

Goeringer GC, Vidic B. 1987. The embryogenesis and anatomy of Waldeyer's ring. Otolaryngol Clin North Am 20(2):207–217.

Gong ZH, Jin HQ, Jin YF, Zhang YZ. 2005. Expression of cholera toxin B subunit and assembly as functional oligomers in silkworm. J Biochem Mol Biol 38(6):717–724.

Groux H, O'Garra A, Bigler M, Rouleau M, Antonenko S, de Vries JE, Roncarolo MG. 1997. A CD4+ T-cell subset inhibits antigen-specific T-cell responses and prevents colitis. Nature 389:737–742.

Guimaraes VC, Quintans J, Fisfalen ME, Straus FH, Wilhelm K, Medeiros-Neto A, DeGroot LJ. 1995. Suppression of development of experimental autoimmune thyroiditis by oral administration of thyroglobulin. Endocrinology 136:3353–3359.

Hancock WW, Polanski M, Zhang J, Blogg N, Weiner HL. 1995. Suppression of insulitis in non-obese diabetic (NOD) mice by oral insulin administration is associated with selective expression of interleukin-4 and -10, transforming growth factor-beta, and prostaglandin-E. Am J Pathol 147:1193–1199.

Haque MA, Yoshino S, Inada S, Nomaguchi H, Tokunaga O, Kohashi O. 1996. Suppression of adjuvant arthritis in rats by induction of oral tolerance to mycobacterial 65-kDa heat shock protein. Eur J Immunol 26:2650–2656.

Harats D, Yacov N, Gilburd B, Shoenfeld Y, George J. 2002. Oral tolerance with heat shock protein 65 attenuates Mycobacterium tuberculosis-induced and high-fat-diet-driven atherosclerotic lesions. J Am Coll Cardiol 40:1333–1338.

Harrison LC. 1992. Islet cell antigens in insulin-dependent diabetes: Pandora's box revisited. Immunol Today 13:348–352.

Harrison LC, Dempsey-Collier M, Kramer DR, Takahashi K. 1996. Aerosol insulin induces regulatory CD8 gamma delta T cells that prevent murine insulin-dependent diabetes. J Exp Med 184 (6):2167–2174.

Higgins PJ, Weiner HL. 1988. Suppression of experimental autoimmune encephalomyelitis by oral administration of myelin basic protein and its fragments. J Immunol 140:440–445.

Holmgren J, Czerkinsky C, Eriksson K, Mharandi A. 2003. Mucosal immunisation and adjuvants: A brief overview of recent advances and challenges. Vaccine 21(Suppl. 2):S89–S95.

Horwitz DA, Zheng SG, Gray JD. 2003. The role of the combination of IL-2 and TGF-beta or IL-10 in the generation and function of CD4+ CD25+ and CD8+ regulatory T cell subsets. J Leukoc Biol 74:471–478.

Inada S, Yoshino S, Haque MA, Ogata Y, Kohashi O. 1997. Clonal anergy is a potent mechanism of oral tolerance in the suppression of acute antigen-induced arthritis in rats by oral administration of the inducing antigen. Cell Immunol 175:67–75.

Inobe J, Slavin AJ, Komagata Y, Chen Y, Liu L, Weiner HL. 1998. IL-4 is a differentiation factor for transforming growth factor-beta secreting Th3 cells and oral administration of IL-4 enhances oral tolerance in experimental allergic encephalomyelitis. Eur J Immunol 28:2780–2790.

Javed NH, Gienapp IE, Cox KL, Whitacre CC. 1995. Exquisite peptide specificity of oral tolerance in experimental autoimmune encephalomyelitis. J Immunol 155:1599–1605.

Karpus WJ, Kennedy KJ, Smith WS, Miller SD. 1996. Inhibition of relapsing experimental autoimmune encephalomyelitis in SJL mice by feeding the immunodominant PLP139-151 peptide. J Neurosci Res 45:410–423.

Kerlero de Rosbo N, Milo R, Lees MB, Burger D, Bernard CC, Ben-Nun A. 1993. Reactivity to myelin antigens in multiple sclerosis. Peripheral blood lymphocytes respond predominantly to myelin oligodendrocyte glycoprotein. J Clin Invest 92:2602–2608.

Khare SD, Krco CJ, Griffiths MM, Luthra HS, David CS. 1995. Oral administration of an immunodominant human collagen peptide modulates collagen-induced arthritis. J Immunol 155:3653–3659.

Khoury SJ, Hancock WW, Weiner HL. 1992. Oral tolerance to myelin basic protein and natural recovery from experimental autoimmune encephalomyelites are associated with down-regulation of inflammatory cytokines and differential upregulation of transforming growth factor b, interleukin 4, and prostraglandin E expression in the brain. J Exp Med 176:1355–1364.

Kim WU, Lee WK, Ryoo JW, Kim SH, Kim J, Youn J, Min SY, Bae EY, Hwang SY, Park SH, Cho CS, Park JS, Kim HY. 2002. Suppression of collagen-induced arthritis by single administration of poly(lactic-co-glycolic acid) nanoparticles entrapping type II collagen: A novel treatment strategy for induction of oral tolerance. Arthritis Rheum 46:1109–1120.

King C, Davies J, Mueller R, Lee MS, Krahl T, Yeung B, O'Connor E, Sarvetnick N. 1998. TGF-beta1 alters APC preference, polarizing islet antigen responses toward a Th2 phenotype. Immunity 8:601–613.

Kurien BT, Asfa S, Li C, Dorri Y, Jonsson R, Scofield RH. 2005. Induction of oral tolerance in experimental Sjogren's syndrome autoimmunity. Scand J Immunol 61(5):418–425.

Lacroix-Desmazes S, Kaveri SV, Mouthon L, Ayouba A, Malanchere E, Coutinho A, Kazatchkine MD. 1998. Self-reactive antibodies (natural autoantibodies) in healthy individuals. J Immunol Methods 216(1–2):117–137.

Lanzavecchia A. 1998. Immunology. Licence to kill. Nature 393:413–414.

Lee S, Scherberg N, DeGroot LJ. 1998. Induction of oral tolerance in human autoimmune thyroid disease. Thyroid 8:229–234.

Lehmann PV, Forsthuber T, Miller A, Sercarz EE. 1992. Spreading of T-cell autoimmunity to cryptic determinants of an autoantigen. Nature 358:155–157.

Li HL, Liu JQ, Bai XF, vn der Meide PH, Link H. 1998. Dose-dependent mechanisms relate to nasal tolerance induction and protection against experimental autoimmune encephalomyelitis in Lewis rats. Immunology. 94(3):431–437.

Liu JQ, Bai XF, Shi FD, Xiao BG, Li HL, Levi M, Mustafa M, Wahren B, Link H. 1998. Inhibition of experimental autoimmune encephalomyelitis in Lewis rats by nasal administration of encephalitogenic MBP peptides: Synergistic effects of MBP 68–86 and 87–99. Int Immunol 10(8):1139–1148.

Lu S, Holmdahl R. 1999. Different therapeutic and bystander effects by intranasal administration of homologous type II and type IX collagens on the collagen-induced arthritis and pristane-induced arthritis in rats. Clin Immunol 90(1):119–127.

Ma CG, Zhang GX, Xiao BG, Link J, Olsson T, Link H. 1995. Suppression of experimental autoimmune myasthenia gravis by nasal administration of acetylcholine receptor. J Neuroimmunol 58(1):51–60.

Ma D, Mellon J, Niederkorn JY. 1998. Conditions affecting enhanced corneal allograft survival by oral immunization. Invest Ophthalmol Vis Sci 39:1835–1846.

Maron R, Palanivel V, Weiner HL, Harn DA. 1998. Oral administration of schistosome egg antigens and insulin β-chain generates and enhances Th2-type responses in NOD mice. Clin Immunol Immunopathol 87:85–92.

Maron R, Sukhova G, Faria AM, Hoffmann E, Mach F, Libby P, Weiner HL. 2002a. Mucosal administration of heat shock protein-65 decreases atherosclerosis and inflammation in aortic arch of low-density lipoprotein receptor-deficient mice. Circulation 106(13):1708–1715.

Maron R, Slavin AJ, Hoffmann E, Komagata Y, Weiner HL. 2002b. Oral tolerance to copolymer 1 in myelin basic protein (MBP) TCR transgenic mice: Cross-reactivity with MBP-specific TCR and differential induction of anti-inflammatory cytokines. Int Immunol 14(2):131–138.

Marth T, Strober W, Kelsall BL. 1996. High dose oral tolerance in Ova TCR-transgenic mice: Systemic neutralization of interleukin 12 augments TGF-b secretion and T cell apoptosis. J Immunol 157:2348–2357.

Masuda K, Horie K, Suzuki R, Yoshikawa T, Hirano K. 2002. Oral delivery of antigens in liposomes with some lipid compositions modulates oral tolerance to the antigens. Microbiol Immunol 46:55–58.

McKown KM, Carbone LD, Bustillo J, Sever JM, Kang AH, Postlethwaite AE. 1997. Open trial of oral type I collagen in patients with systemic sclerosis. Arthritis Rheum 40:S100.

McKown KM, Carbone LD, Bustillo J, Seyer JM, Kang AH, Postlethwaite AE. 2000. Induction of immune tolerance to human type I collagen in patients with systemic sclerosis by oral administration of bovine type I collagen. Arthritis Rheum 43:1054–1061.

Melamed D, Friedman A. 1993. Direct evidence for anergy in T lymphocytes tolerized by oral administration of ovalbumin. Eur J Immunol 23:935–942.

Mengel J, Favaro P, Meyer A, Motta V, de Alencar R, Postol E, Cardillo F. 2005. Potentiation of immunological tolerance induction in adult mice by co-administration of pooled normal IgG and oral tolerogens: A potential therapeutic approach for autoimmune diseases. Med Hypotheses 64(5):978–985.

Metzler B, Wraith DC. 1993. Inhibition of experimental autoimmune encephalomyelitis by inhalation but not oral administration of the encephalitogenic peptide: Influence of MHC binding affinity. Int Immunol 5(9):1159–1165.

Meyer A, Gienapp I, Cox K, Goverman J, Hood L, Whitacre C. 1996. Oral tolerance in myelin basic protein TCR transgenic mice. Ann N Y Acad Sci 778:412–413.

Meyer AL, Benson J, Song F, Javed N, Gienapp IE, Goverman J, Brabb TA, Hood L, Whitacre CC. 2001. Rapid depletion of peripheral antigen-specific T cells in TCR-transgenic mice after oral administration of myelin basic protein. J Immunol 166:5773–5781.

Miller A, Lider O, Weiner HL. 1991. Antigen driven bystander suppression after oral administration of antigen. J Exp Med 174:791–798.

Miller A, Lider O, Roberts AB, Sporn MB, Weiner HL. 1992. Suppressor T cells generated by oral tolerization to myelin basic protein suppress both *in vivo* and *in vitro* immune response by the release of transforming growth factor beta after antigen-specific triggering. Proc Natl Acad Sci USA 89:421–425.

Miller A, Zhang ZJ, Sobel RA, al-Sabbagh A, Weiner HL. 1993. Suppression of experimental autoimmune encephalomyelitis by oral administration of myelin basic protein. VI. Suppression of adoptively transfered disease and differential effects of oral vs. intravenous tolerization. J Neuroimmunol 46:73–82.

Modigliani Y, Coutinho A, Pereira P, Le Douarin N, Thomas-Vaslin V, Burlen-Defranoux O, Salaun J, Bandeira A. 1996. Establishment of tissue-specific tolerance is driven by regulatory T cells selected by thymic epithelium. Eur J Immunol August 26(8):1807–1815.

Monetini L, Cavallo MG, Sarugeri E, Sentinelli F, Stefanini L, Bosi E, Thorpe R, Pozzilli P. 2004. Immunotherapy Diabetes (IMDIAB) group. Cytokine profile and insulin antibody IgG subclasses in patients with recent onset type 1 diabetes treated with oral insulin. Diabetologia 47(10):1795–1802.

Monsonego A, Beserman ZP, Kipnis J, Yoles E, Weiner HL, Schwartz M. 2003. Beneficial effect of orally administered myelin basic protein in EAE-susceptible Lewis rats in a model of acute CNS degeneration. J Autoimmun 21:131–138.

Mowat AM, Strobel S, Drummond HE, Ferguson A. 1982. Immunological response to fed protein antigens in mice. I. Reversal of oral tolerance to ovalbumin by cyclophosphamide. Immunology 45:105–113.

Mucida D, Kutchukhidze N, Erazo A, Russo M, Lafaille JJ, Curotto de Lafaille MA. 2005. Oral tolerance in the absence of naturally occurring Tregs. J Clin Invest 115(7):1923–1933.

Myers LK, Seyer JM, Stuart JM, Kang AH. 1997. Suppression of murine collagen-induced arthritis by nasal administration of collagen. Immunology 90(2):161–164.

Myers LK, Higgins GC, Finkel TH, Reed AM, Thompson JW, Walton RC, Hendrickson J, Kerr NC, Pandya-Lipman RK, Shlopov BV, Stastny P, Postelthwaite AE, Kang AH. 2001. Juvenile arthritis and autoimmunity to type II collagen. Arthritis Rheum 44(8):1775–1781.

Nagatani K, Sagawa K, Komagata Y, Yamamoto K. 2004. Peyer's patch dendritic cells capturing oral antigen interact with antigen-specific t cells and induce gut-homing CD4+CD25+ regulatory T Cells in Peyer's patches. Ann N Y Acad Sci 1029: 366–370.

Nagler-Anderson C, Bober LA, Robinson ME, Siskind GW, Thorbecke JG. 1986. Suppression of type II collagen-induced arthritis by intragastric administration of soluble type II collagen. Proc Nat Acad Sci USA 83:7443–7446.

Nakamura K, Kitani A, Strober W. 2001. Cell contact-dependent immunosuppression by CD4(+)CD25(+) regulatory T cells is mediated by cell surface-bound transforming growth factor beta. J Exp Med 194:629–644.

Nakamura K, Kitani A, Fuss I, Pedersen A, Harada N, Nawata H, Strober W. 2004. TGF-beta 1 plays an important role in the mechanism of CD4+CD25+ regulatory T cell activity in both humans and mice. J Immunol 172:834–842.

Nelson PA, Akselband Y, Dearborn SM, Al-Sabbagh A, Tian ZJ, Gonnella PA, Zamvil SS, Chen Y, Weiner HL. 1996. Effect of oral beta interferon on subsequent immune responsiveness. Ann N Y Acad Sci 778:145–155.

Neurath MF, Fuss I, Kelsall BL, Presky DH, Waegell W, Strober W. 1996. Experimental granulomatous colitis in mice is abrogated by induction of TGF-beta-mediated oral tolerance. J Exp Med 183:2605–2616.

Nussenblatt RB, Caspi RR, Mahdi R, Chaochau C, Roberge F, Lide O, Weiner HL. 1990. Inhibition of S antigen induced experimental autoimmune uveoretinitis by oral induction of tolerance with S antigen. J Immunol 144:1689–1695.

Nussenblatt RB, Gery I, Weiner HL, Ferris FL, Shiloach J, Remaley N, Perry C, Caspi RR, Hafler DA, Foster CS, Whitcup SM. 1997. Treatment of uveitis by oral administration of retinal antigens: Results of a phase I/II randomized masked trial. Am J Ophthalmol 123:583–592.

Oida T, Zhang X, Goto M, Hachimura S, Totsuka M, Kaminogawa S, Weiner HL. 2003. CD4+CD25− T cells that express latency-associated peptide on the surface suppress CD4+-CD45RBhigh-induced colitis by a TGF-beta-dependent mechanism. J Immunol 170:2516–2522.

Okumura S, McIntosh K, Drachman DB. 1994. Oral administration of acetylcholine receptor: Effects on experimental myasthenia gravis. Ann Neurol 36:704–713.

Ostroukhova M, Seguin-Devaux C, Oriss TB, Dixon-McCarthy B, Yang L, Ameredes BT, Corcoran TE, Ray A. 2004. Tolerance induced by inhaled antigen involves CD4(+) T cells expressing membrane-bound TGF-beta and FOXP3. J Clin Invest 114(1):28–38.

Paul WE, Seder RA. 1994. Lymphocyte responses and cytokines. Cell 76:241–251.

Pecquet S, Leo E, Fritsche R, Pfeifer A, Couvreur P, Fattal E. 2000. Oral tolerance elicited in mice by β-lactoglobulin entrapped in biodegradable microspheres. Vaccine 18:1196–1202.

Peterson KE, Braley-Mullen H. 1995. Suppression of murine experimental autoimmune thyroiditis by oral administration of porcine thyroglobulin. Cell Immunol 166:123–130.

Polanski M, Melican NS, Zhang J, Weiner HL. 1997. Oral administration of the immunodominant B-chain of insulin reduces diabetes in a co-transfer model of diabetes in the NOD mouse and is associated with a switch from Th1 to Th2 cytokines. J Autoimmun 10:339–346.

Postlethwaite AE. 2001. Can we induce tolerance in rheumatoid arthritis? Curr Rheumatol Rep 3:64–69.

Ramachandran A, Madesh M, Balasubramanian KA. 2000. Apoptosis in the intestinal epithelium: Its relevance in normal and pathophysiological conditions. J Gastroenterol Hepatol 15:109–120.

Rizzo LV, Miller-Rivero NE, Chan CC, Wiggert B, Nussenblatt RB, Caspi RR. 1994. Interleukin-2 treatment potentiates induction of oral tolerance in a murine model of autoimmunity. J Clin Invest 94:1668–1672.

Sakaguchi S. 2004. Naturally arising CD4+ regulatory t cells for immunologic self-tolerance and negative control of immune responses. Annu Rev Immunol 22:531–562.

Samsom JN, Hauet-Broere F, Unger WW, VAN Berkel LA, Kraal G. 2004. Early events in antigen-specific regulatory T cell induction via nasal and oral mucosa. Ann N Y Acad Sci 1029:385–389.

Santos LM, al-Sabbagh A, Londono A, Weiner HL. 1994. Oral tolerance to myelin basic protein induces regulatory TGF-beta-secreting T cells in Peyer's patches of SJL mice. Cell Immunol 157:439–447.

Shi FD, Li H, Wang H, Bai X, van der Meide PH, Link H, Ljunggren HG. 1999. Mechanisms of nasal tolerance induction in experimental autoimmune myasthenia gravis: Identification of regulatory cells. J Immunol 162(10):5757–5763.

Shi HN, Liu HY, Nagler-Anderson C. 2000. Enteric infection acts as an adjuvant for the response to a model food antigen. J Immunol 165:6174–6182.

Simioni PU, Fernandes LG, Gabriel DL, Tamashiro WM. 2004. Induction of systemic tolerance in normal but not in transgenic mice through continuous feeding of ovalbumin. Scand J Immunol 60(3):257–266.

Slavin AJ, Maron R, Weiner HL. 2001. Mucosal administration of IL-10 enhances oral tolerance in autoimmune encephalomyelitis and diabetes. Int Immunol 13:825–833.

Soos JM, Stuve O, Youssef S, Bravo M, Johnson HM, Weiner HL, Zamvil SS. 2002. Cutting edge: Oral type I IFN-tau promotes a Th2 bias and enhances suppression of autoimmune encephalomyelitis by oral glatiramer acetate. J Immunol 169:2231–2235.

Staines NA, Harper N, Ward FJ, Malmstrom V, Holmdahl R, Bansal S. 1996. Mucosal tolerance and suppression of collagen-induced arthritis (CIA) induced by nasal inhalation of synthetic peptide 184–198 of bovine type II collagen (CII)expressing a dominant T cell epitope. Clin Exp Immunol 103(3):368–375.

Sugihara R, Matsumoto Y, Ohmori H. 2002. Suppression of IgE antibody response in mice by a polysaccharide, AZ9, produced by Klebsiella oxytoca strain TNM3. Immunopharmacol Immunotoxicol 24:245–254.

Taams LS, Vukmanovic-Stejic M, Smith J, Dunne PJ, Fletcher JM, Plunkett FJ, Ebeling SB, Lombardi G, Rustin MH, Bijlsma JW, Lafeber FP, Salmon M, Akbar AN. 2002. Antigen-specific T cell suppression by human CD4+CD25+ regulatory T cells. Eur J Immunol 32:1621–1630.

Takahashi T, Kuniyasu Y, Toda M, Sakaguchi N, Itoh M, Iwata M, Shimizu J, Sakaguchi S. 1998. Immunologic self-tolerance maintained by CD25+CD4+ naturally anergic and suppressive T cells: Induction of autoimmune disease by breaking their anergic/suppressive state. Int Immunol 10:1969–1980.

Teitelbaum D, Arnon R, Sela M. 1999. Immunomodulation of experimental autoimmune encephalomyelitis by oral administration of copolymer 1. Proc Natl Acad Sci USA 96:3842–3847.

Thompson HS, Staines NA. 1986. Prevention of collagen-induced arthritis by oral administration of encapsulated type II collagen. Clin Exp Immunol 64:581–586.

Thompson HS, Harper N, Bevan DJ, Staines NA. 1993. Suppression of collagen induced arthritis by oral administration of type II collagen: Changes in immune and arthritic responses mediated by active peripheral suppression. Autoimmunity 16: 189–199.

Thorbecke GJ, Schwarcz R, Leu J, Huang C, Simmons WJ. 1999. Modulation by cytokines of induction of oral tolerance to type II collagen. Arthritis Rheum 42:110–118.

Thurau SR, Chan CC, Nussenblatt RB, Caspi RR. 1997a. Oral tolerance in a murine model of relapsing experimental autoimmune uveoretinitis (EAU): Induction of protective tolerance in primed animals. Clin Exp Immunol 109:370–376.

Thurau SR, Diedrichs-Mohring M, Fricke H, Arbogast S, Wildner G. 1997b. Molecular mimicry as a therapeutic approach for an autoimmune disease: Oral treatment of uveitis-patients with an MHC-peptide crossreactive with autoantigen-first results. Immunol Lett 57:193–201.

Tian J, Atkinson MA, Clare-Salzler M, Herschenfeld A, Forsthuber T, Lehmann PV, Kaufman DL. 1996. Nasal administration of glutamate decarboxylase (GAD65) peptides induces Th2 responses and prevents murine insulin-dependent diabetes. J Exp Med 183(4):1561–1567.

Tisch R, Yang XD, Singer SM, Liblau RS, Fugger L, McDevitt HO. 1993. Immune response to glutamic acid decarboxylase correlates with insulitis in non-obese diabetic mice. Nature 366:72–75.

Torseth JW, Gregerson DS. 1998. Oral tolerance in experimental autoimmune uveoretinitis: Feeding after disease induction is less protective than prefeeding. Clin Immunol Immunopathol 88:297–304.

Trentham DE, Dynesius-Trentham RA, Orav EJ, Combitchi D, Lorenzo C, Sewell KL, Hafler DA, Weiner HL. 1993. Effects of oral administration of type II collagen on rheumatoid arthritis. Science 261:1727–1730.

Trentham DE, Townes AS, Kang AH. 1977. Autoimmunity to type II collagen an experimental model of arthritis. J Exp Med 146:857–868.

van Eden W, Thole JE, van der Zee R, Noordzij A, van Embden JD, Hensen EJ, Cohen IR. 1988. Cloning of the mycobacterial epitope recognized by T lymphocytes in adjuvant arthritis. Nature 331:171–173.

Vaz NM, Maia LC, Hanson DG, Lynch JM. 1977. Inhibition of homocytotropic antibody responses in adult inbred mice by previous feeding of the specific antigen. J Allergy Clin Immunol 60(2):110–115.

Vaz NM, Maia LC, Hanson DG, Lynch JM. 1981. Cross-suppression of specific immune responses after oral tolerance. Mem Inst Oswaldo Cruz 76(1):83–91.

Viney JL, Mowat AM, O'Malley JM, Williamson E, Fanger NA. 1998. Expanding dendritic cells *in vivo* enhances the induction of oral tolerance. J Immunol 160(12):5815–5825.

von Herrath MG, Dyrberg T, Oldstone MB. 1996. Oral insulin treatment suppresses virus-induced antigen-specific destruction of beta cells and prevents autoimmune diabetes in transgenic mice. J Clin Invest 98:1324–1331.

Wang ZY, Qiao J, Link H. 1993. Suppression of experimental autoimmune myasthenia gravis by oral administration of acetylcholine receptor. J Neuroimmunol 44:209–214.

Wang ZY, Link H, Ljungdahl A, Hojeberg B, Link J, He B, Qiao J, Melms A, Olsson T. 1994. Induction of interferon-gamma, interleukin-4, and transforming growth factor-beta in rats orally tolerized against experimental autoimmune myasthenia gravis. Cell Immunol 157:353–368.

Wang ZY, He B, Qiao J, Link H. 1995. Suppression of experimental autoimmune myasthenia gravis and experimental allergic encephalomyelitis by oral administration of acetylcholine receptor and myelin basic protein: Double tolerance. J Neuroimmunol 63(1):79–86.

Weiner HL. 1999. Oral tolerance with copolymer 1 for the treatment of multiple sclerosis. Proc Natl Acad Sci USA 96:3333–3335.

Weiner HL. 2001. The mucosal milieu creates tolerogenic dendritic cells and T(R)1 and T(H)3 regulatory cells. Nat Immunol 2: 671–672.

Weiner HL, Komagata Y. 1998. Oral tolerance and the treatment of rheumatoid arthritis. Springer Semin Immunopathol 20: 289–308.

Whitacre CC, Gienapp IE, Orosz CG, Bitar DM. 1991. Oral tolerance in experimental autoimmune encephalomyelites. III. Evidence for clonal anergy. J Immunol 147: 2155–2163.

Wildner G, Thurau SR. 1994. Cross-reactivity between an HLA-B27-derived peptide and a retinal autoantigen peptide: A clue to major histocompatibility complex association with autoimmune disease. Eur J Immunol 24:2579–2585.

Yoshino S. 1995. Antigen-induced arthritis in rats is suppressed by the inducing antigen administered orally before, but not after immunization. Cell Immunol 163:55–58.

Zhang W, Kong YC. 1998. Noninvolvement of IL-4 and IL-10 in tolerance induction to experimental autoimmune thyroiditis. Cell Immunol 187:95–102.

Zhang Z, Michael JG. 1990a. Orally inducible immune responsiveness is abrogated by IFN-τ treatment. J Immunol 144: 4163–4165.

Zhang JA, Lee CSY, Lider O, Weiner JM. 1990b. Suppression of adjuvant arthritis in Lewis rats by oral administration of type II collagen. J Immunol 145:2489–2493.

Zhang JZ, Lee CSY, Lider O, Weiner HL. 1991. Suppression of diabetes in NOD mice by oral administration of procine insulin. Proc Natl Acad Sci USA 88:10252–10256.

Zhang J, Markovic-Plese S, Lacet B, Raus J, Weiner HL, Hafler DA. 1994. Increased frequency of interleukin 2-responsive T cells specific for myelin basic protein and proteolipid protein in peripheral blood and cerebrospinal fluid of patients with multiple sclerosis. J Exp Med 179:973–984.

Zhang X, Izikson L, Liu L, Weiner HL. 2001. Activation of CD25(+)CD4(+) regulatory T cells by oral antigen administration. J Immunol 167:4245–4253.

Zhou P, Borojevic R, Streutker C, Snider D, Liang H, Croitoru K. 2004. Expression of dual TCR on DO11.10 T cells allows for ovalbumin-induced oral tolerance to prevent T cell-mediated colitis directed against unrelated enteric bacterial antigens. J Immunol 172:1515–1523.

Zhu J, Deng GM, Levi M, Wahren B, Diab A, van der Meide PH, Link H. 1998. Prevention of experimental autoimmune neuritis by nasal administration of P2 protein peptide 57–81. J Neuropathol Exp Neurol 57(3):291–301.

